# Normal Hematopoietic Stem Cells in Leukemic Bone Marrow Environment Undergo Morphological Changes Identifiable by Artificial Intelligence

**DOI:** 10.3390/ijms262110354

**Published:** 2025-10-24

**Authors:** Dongguang Li, Athena Li, Ngoc DeSouza, Shaoguang Li

**Affiliations:** 1 Division of Hematology/Oncology, Department of Medicine, University of Massachusetts Chan Medical School, Worcester, MA 01605, USA; dongguang.li1@umassmed.edu (D.L.); ngoc.desouza@umassmed.edu (N.D.); 2 School of Arts and Sciences, Tufts University, Medford, MA 02155, USA; liathena8@gmail.com

**Keywords:** hematopoietic stem cells, leukemia stem cells, artificial intelligence, polycythemia vera

## Abstract

Leukemia stem cells (LSCs) in numerous hematologic malignancies are generally believed to be responsible for disease initiation, progression/relapse and resistance to chemotherapy. It has been shown that non-leukemic hematopoietic cells are affected molecularly and biologically by leukemia cells in the same bone marrow environment where both non-leukemic hematopoietic stem cells (HSCs) and LSCs reside. We believe the molecular and biological changes of these non-leukemic HSCs should be accompanied by the morphological changes of these cells. On the other hand, the quantity of these non-leukemic HSCs with morphological changes should reflect disease severity, prognosis and therapy responses. Thus, identification of non-leukemic HSCs in the leukemia bone marrow environment and monitoring of their quantity before, during and after treatments will potentially provide valuable information for correctly handling treatment plans and predicting outcomes. However, we have known that these morphological changes at the stem cell level cannot be extracted and identified by microscopic visualization with human eyes. In this study, we chose polycythemia vera (PV) as a disease model (a type of human myeloproliferative neoplasms derived from a hematopoietic stem cell harboring the *JAK2V617F* oncogene) to determine whether we can use artificial intelligence (AI) deep learning to identify and quantify non-leukemic HSCs obtained from bone marrow of *JAK2V617F* knock-in PV mice by analyzing single-cell images. We find that non-*JAK2V617F*-expressing HSCs are distinguishable from LSCs in the same bone marrow environment by AI with high accuracy (>96%). More importantly, we find that non-*JAK2V617F*-expressing HSCs from the leukemia bone marrow environment of PV mice are morphologically distinct from normal HSCs from a normal bone marrow environment of normal mice by AI with an accuracy of greater than 98%. These results help us prove the concept that non-leukemic HSCs undergo AI-recognizable morphological changes in the leukemia bone marrow environment and possess unique morphological features distinguishable from normal HSCs, providing a possibility to assess therapy responses and disease prognosis through identifying and quantitating these non-leukemic HSCs in patients.

## 1. Introduction

Leukemia stem cells (LSCs) are thought to be closely related to disease initiation/progression, relapse and drug resistance/insensitivity in numerous types of hematologic malignancies [1,2,3]. On the other hand, LSCs not only proliferate and differentiate to give rise to full bloom of leukemia but also create a leukemic microenvironment that negatively affects the functions of their surrounding normal hematopoietic stem cells (HSCs) [4]. We believe that the functional changes of normal HSCs in the leukemic bone marrow environment accompany with morphological (structural) changes of these cells, and these changes should be recognizable, thereby presenting new morphological features that can be used for specifically identifying these cells. Moreover, we believe that identification and quantitation of these normal HSCs in the leukemic bone marrow environment could help to evaluate leukemia severity, therapy response and disease outcome. Therefore, there is an unmet need to develop an accurate method to monitor the levels of normal HSCs in patients.

In our previous studies, we developed an accurate artificial intelligence (AI) deep learning method to specifically identify and quantitate LSCs and distinguish them from their normal stem cell counterparts as well as other leukemia and normal hematopoietic lineages [5], and we used AI because different morphological features of various types of stem and progenitor cells could not be recognized by microscopic visualization by human eyes. Thus, it is likely that we will be able to identify and extract morphological features from normal HSCs by AI deep learning, which has not been proposed and achieved by any other research groups so far. Although flow cytometry is often used for identifying normal HSCs and other hematopoietic cells based on cell surface markers, it does not distinguish normal HSCs from LSCs, because these cell surface markers are often expressed on both normal HSCs and LSCs [6,7]. Again, we believe that normal HSCs in the leukemia bone marrow environment undergo morphological changes, but these changes are difficult to recognize by human visualization. Here we prove the concept that the morphological changes of normal HSCs in the leukemia bone marrow environment can be recognized by AI deep learning. Based on our recent successes in using AI to read cell or tissue images for disease diagnosis [8], we are confident that AI deep learning will allow extract and identify the morphological features presented by HSCs for distinguishing them from normal stem cell counterparts. Polycythemia vera (PV) is a common form of human myeloproliferative neoplasm derived from an LSC, thereby serving as an ideal model disease for training and testing AI models to help to identify and quantitate normal HSCs in PV mice.

## 2. Results

### 2.1. Molecular Changes in LSCs Reflect Cellular Functions

**Figure 1:**
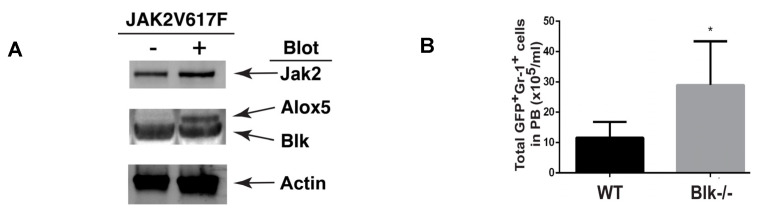
JAK2V617F downregulates Blk and upregulates Alox5, and *Blk* deficiency favors PV development. (**A**) Non-JAK2V617F-expressing and JAK2V617F-expressing Ba/F3 cells were starved in serum-free medium overnight and then cultured in the presence of serum for 24 h. Protein lysates were analyzed by Western blot using antibodies against Jak2 (for detecting JAK2V617F), Blk, Alox5, and beta-actin (protein loading control). (**B**) Bone marrow cells from wild type (WT) or *Blk*^−/−^ mice were transduced with *JAK2V617F*-*GFP* retrovirus, followed by transplantation into lethally irradiated WT recipient mice to induce PV. Two weeks later, GFP^+^Gr-1^+^ cells in peripheral blood (PB) of the mice were analyzed by FACS (*p* < 0.01 *).

**Figure 2:**
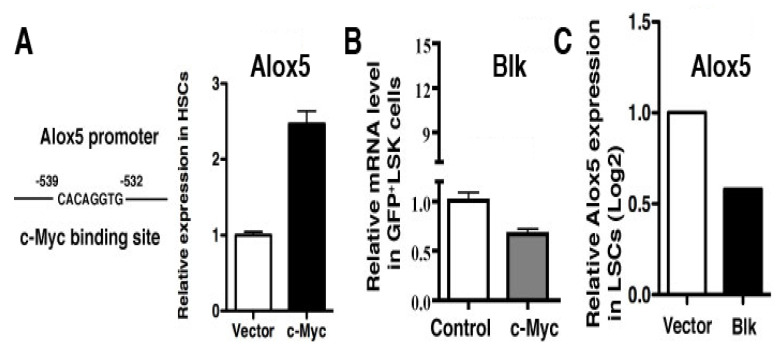
*c-Myc* regulates *Alox5* expression through *Blk*. (**A**) *c-Myc* has a binding site on the *Alox5* promoter and stimulates *Alox5* expression in HSCs. BM cells transduced with GFP vector or *c-Myc*-GFP were transplanted into mice. After 14 days, HSCs (Lin*^−^*Sca-1^+^c-Kit^+^, LSK) were sorted from BM by FACS for RT-PCR analysis of *Alox5* expression. (**B**) RT-PCR detection of *Blk* in HSCs transduced with *c-Myc* lentivirus. (**C**) DNA microarray shows a down-regulation of *Alox5* by *Blk* in LSCs.

**Figure 3:**
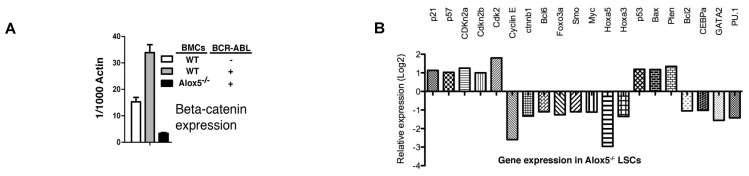
Genes in the *Alox5* pathway. (**A**) Beta-catenin is a downstream gene of *Alox5*. Bone marrow GFP^+^Lin*^−^*c-Kit^+^Sca-1^+^ cells were sorted by FACS from recipients of GFP vector- or BCR-ABL-transduced bone marrow cells from wild type or *Alox^−/−^* donor mice for isolation of total RNA. Expression of *beta-catenin* was detected by RT-PCR. (**B**) Gene expression changes between wild type and *Alox5^−/−^* LSCs. BM cells from C57BL/6 (B6) or *Alox5^−/−^* mice were transduced with *BCR-ABL-GFP*, and then transferred into B6 mice. At day 14 post BM transplantation, BM cells from CML mice were sorted by FACS for GFP^+^Lin^−^Sca-1^+^c-Kit^+^cells, and total RNA was isolated for DNA micorarray analysis. The significantly altered genes associated with apoptosis, cell cycle control and proliferation of cells are listed.

Because different types of leukemia are driven by different types of oncogenes such as *JAK2V617F* [9,10,11,12] vs. BCR-ABL [13,14], we thought that these oncogenes function by activating or inhibiting different groups of genes, although some genes can be shared. Thus, we believe that the morphology of one type of LSCs should be different from that of another type of LSCs, which would provide a basis for distinguishing them from each other by identifying and extracting morphological features from these cells. To collect evidence that supports this idea, we considered our previous findings that the Alox5 gene plays an essential role in functional regulation of LSCs for both PV and CML [15,16], two different types of myeloproliferative neoplasms driven by *JAK2V617F* and *BCR-ABL*, respectively. On the one hand, JAK2V617F downregulated expression of Blk (B lymphocyte kinase) in JAK2V617F-expressing Ba/F3 cells (Figure 1A) and upregulated expression of Alox5 in PV mice (Figure 1B), and on the other hand, BCR-ABL also upregulates Alox5 expression [15] and did so through *c-Myc* (Figure 2A) and *Blk* (Figure 2B,C). In other words, Blk is involved in signaling by both JAK2V617F and BCR-ABL in two different types of myeloid malignancies (Figure 1 and Figure 2). Furthermore, we found that *BCR-ABL* activates *beta-catenin* (Figure 3A) and a group of other genes (Figure 3B) through *Alox5*. These results enforced our belief that molecular changes in LSCs reflect cellular functions, prompting us to develop a method to morphologically identify LSCs and other hematopoietic cells affected by LSCs in the same leukemic bone marrow environment.

### 2.2. Normal Hematopoietic Stem Cells (HSCs) in the Leukemic Bone Marrow Environment Undergo Morphological Changes

**Figure 4:**
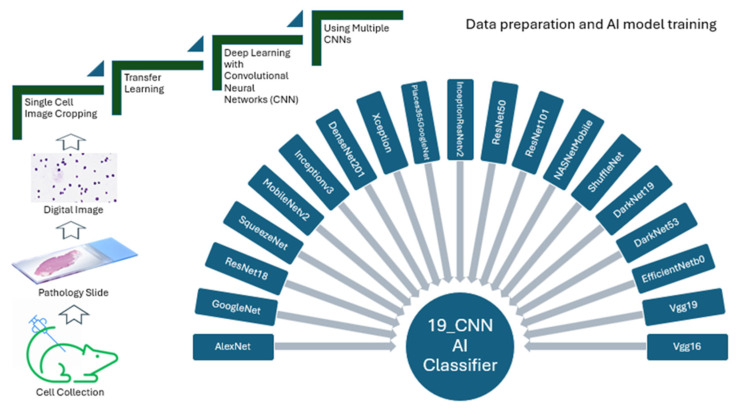
Flowchart of our cell image preparation procedure and AI deep learning method with multiple CNNs. Bone marrow cells were collected from mouse bone marrow to prepared single-cell images stained with Wright and Giemsa for further scanning and analysis. Transfer learning was employed during AI model building using multiple CNNs (19 CNNs in this study).

It has been shown that normal hematopoietic cells are biologically affected by leukemia cells in the bone marrow environment where both leukemic and normal stem cells reside [17,18]. Therefore, we hypothesized that the morphological features of normal long-term HSCs (LT-HSCs) are changed in the same leukemic bone marrow environment where LSCs also reside. To test this hypothesis, we use multiple pretrained deep neural networks to classify the content of single-cell images for the best performance in classifications. In this study, we combined 19 CNNs (convolutional neural networks) together (Table 1) in building our AI models for analysis of single-cell images (Figure 4). We took the multiple-CNN approach in building AI models because our previous work suggests that this multiple-CNN approach led to achieving a higher classification accuracy than the single CNN approach did [8].

**Figure 5:**
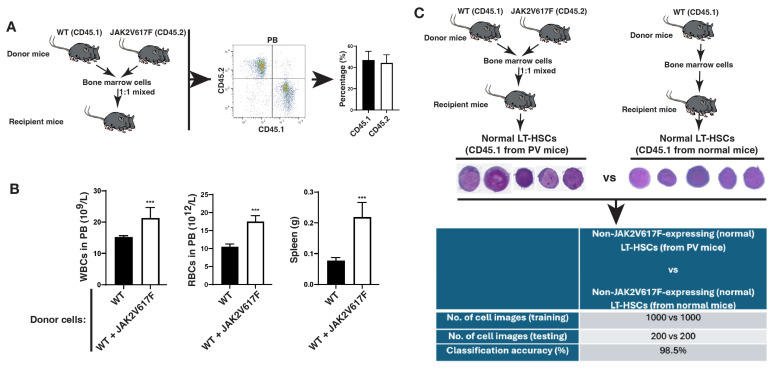
Normal hematopoietic stem cells (HSCs) in the leukemic bone marrow environment undergo morphological changes identifiable by AI deep learning. (**A**) Bone marrow cells from JAK2V617F knock-in mice (CD45.2) and normal bone marrow cells from wild type mice (CD45.1) were 1:1 mixed and then transplanted into lethally irradiated recipient mice (2 × 10^6^ bone marrow cells per mouse for each type of donor cells). Six weeks after bone marrow transplantation, peripheral blood cells of the recipient mice were analyzed by FACS for the presence of both CD45.2 and CD45.1 cells at a similar percentage. (**B**) White blood cells (WBCs), red blood cells (RBCs) and spleens from the recipient mice receiving 1:1 mixed JAK2 mutant (JAK2V617F) and wild-type bone marrow cells were analyzed six weeks after the transplantation to show the development of myeloproliferative disease in the mice. Wild-type recipient mice (CD45.2) receiving wild type donor bone marrow cells (CD45.1) were used as control. (**C**) Six weeks after the transplantation, normal [non-JAK2V617F-expressing LT-HSCs (CD45.1)] bone marrow cells from the recipient mice that developed PV as shown in (**A**,**B**) were isolated and sorted into LT-HSCs. After W&G staining, individual cell images (400×) were scanned for training and testing our AI models, compared to normal LT-HSCs sorted from bone marrow of recipient mice receiving wild-type bone marrow cells (CD45.1). A high classification accuracy (98.5%) was achieved in identifying normal HSCs in leukemic and normal bone marrow environment and distinguishing them from each other. ***: *p* < 0.01.

In mice, normal HSCs reside in the LSK (Lin^−^Sca-1^+^c-Kit^+^) cell population in bone marrow, and LSK cells are composed of three populations: LT-HSCs (long-term HSCs), ST-HSCs (short-term HSCs) and MPPs (multipotent progenitors). To test our hypothesis described above, we focused on JAK2V617F-expressing LT-HSCs collected from bone marrow of JAK2V617F knock-in mice (Jak-Cre) [19], and compared them with normal LT-HSCs collected from bone marrow of the same PV mice. Thus, JAK2V617F-expressing LT-HSCs and non-JAK2V617F-expressing LT-HSCs resided in the same bone marrow environment of the same PV mice. In detail, we mixed donor bone marrow cells from JAK2V617F knock-in mice (CD45.2) with normal bone marrow cells from wild-type (WT) mice (CD45.1) at a 1:1 ratio and then transplanted the mixed cells into the same recipient mice to allow co-existence of JAKV617F-expressing and non-JAK2V617F-expressing (normal) cells in the same bone marrow environment of a recipient mouse. Six weeks after the transplantation, a similar percentage of CD45.2 and CD45.1 white blood cells was detected in each recipient mouse (Figure 5A). These recipient mice developed PV as shown by increased white blood cell counts, red blood cell counts, and spleen weight (Figure 5B). Bone marrow cells were isolated from the recipient mice with PV for sorting of normal LT-HSCs (CD45.1^+^Lin^−^Sca-1^+^c-Kit^+^CD150^+^CD48^−^), and for comparison, normal LT-HSCs (CD45.1) from normal mice were isolated from recipient mice receiving WT donor bone marrow cells (Figure 5C). Single-cell images were prepared and stained with Wright and Giemsa (W&G) for morphological analysis. There were no distinguishable morphological features identified by microscopic visualization by human eyes between normal LT-HSCs from PV mice and those from normal mice (Figure 5C), prompting us to test whether we could use AI deep learning to identify and extract the morphological features altered by the leukemic bone marrow environment. To do so, we trained and validated our AI model using 1000 single-cell images from each group and used the remaining 200 single-cell images to test the AI model. We found that our AI models achieved greater than 98% classification accuracy for distinguishing between the two groups (Figure 5C), demonstrating that normal HSCs in the leukemic bone marrow environment undergo morphological changes that can be identified by AI deep learning but not by human eyes under a microscope.

### 2.3. The LSC Population Is Morphologically Distinguishable from Its Normal Stem Cell Counterpart in the Same Bone Marrow Environment

**Figure 6:**
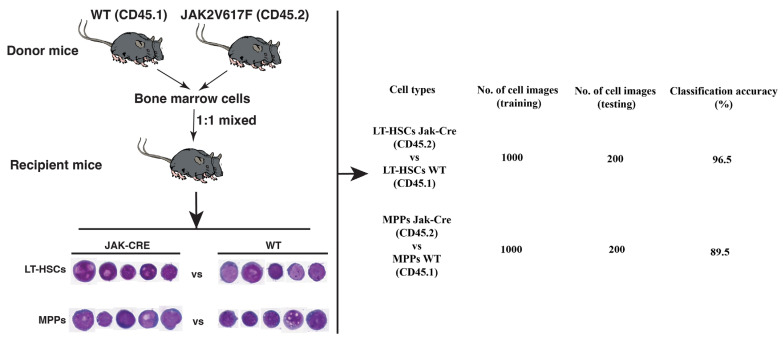
LSCs and their normal stem cell counterparts in the same bone marrow environment can be distinguished morphologically by AI deep learning. Bone marrow cells from *JAK2V617F* knock-in mice (*JAK-CRE*, CD45.2) and normal bone marrow cells from wild type mice (WT, CD45.1) were 1:1 mixed and then transplanted into lethally irradiated recipient mice (2 × 10^6^ bone marrow cells per mouse for each type of donor cells). Six weeks after bone marrow transplantation, bone marrow cells from the recipient mice were isolated and sorted into four cell groups: *JAK2V617F*-expressing LT-HSCs (CD45.2), *JAK2V617F*-positive MPPs (CD45.2), wild type LT-HSCs (CD45.1), and wild type MPPs (CD45.1). Individual cell images (400×) were scanned for training and testing our AI models that showed high accuracy in distinguishing *JAK2V617F*-expressing LT-HSCs and MPPs from wild type LT-HSCs and MPPs.

In PV, normal HSCs acquire the *JAK2V617F* mutation to become LSCs [20]. We believe that the acquisition of *JAK2V617F* not only transforms normal HSCs to LSCs but also causes morphological changes of the cells, providing the conceptual basis for morphologically distinguishing LSCs from their normal stem cell counterparts. If leukemic environment causes morphological changes in normal HSCs (Figure 5), we wondered whether these normal HSCs are morphologically distinguishable from LSCs in the same bone marrow environment. We focused on two types of cells in the LSC population and normal stem cell population: LT-HSCs and MPPs (multipotent progenitors). As shown in Figure 5, we mixed bone marrow cells from *JAK2V617F* transgenic mice (CD45.2) with normal bone marrow cells from wild-type mice (CD45.1) and then transplanted the mixed cells into the same recipient mice to allow co-existence of *JAKV617F*-expressing and non-*JAK2V617F*-expressing (normal) cells in the same bone marrow environment of a recipient mouse. Six weeks after the transplantation, bone marrow cells were isolated from the recipient mice and sorted into four primitive cell groups: JAK2V617F-expressing LT-HSCs (CD45.2^+^Lin^−^Sca-1^+^c-Kit^+^CD150^+^CD48^−^), JAK2V617F-expressing MPPs (CD45.2^+^Lin^−^Sca-1^+^c-Kit^+^CD150^−^CD48^+^), wild type LT-HSCs (CD45.1^+^Lin^−^Sca-1^+^c-Kit^+^CD150^+^CD48^−^), and wild-type MPPs (CD45.1^+^Lin^−^Sca-1^+^c-Kit^+^CD150^−^CD48^+^). Analysis of single-cell images of these cells showed that our AI models achieved close to 97% classification accuracy for distinguishing between JAK2V617F-positive and normal LT-HSCs and 90% classification accuracy for distinguishing between JAK2V617F-positive and normal MPPs (Figure 6).

## 3. Discussion

Although it is commonly agreed that leukemic cells have a strong negative effect on non-leukemic (normal) cells in the same bone marrow environment, there have been no available methods allowing for specifically identifying and visualizing normal hematopoietic stem cell populations in hematologic malignancies microscopically. In this study, we were able to use AI deep learning to identify and extract the morphological features of normal and leukemic stem cells in the same bone marrow environment to distinguish them from each other. More importantly, we were able to prove the concept, based on our study using PV knock-in mice, that the effect of leukemic bone marrow environment on normal stem cells can be reflected by the morphological changes that can be identified by our AI deep learning method. In other words, we propose for the first time that normal stem cells in a leukemic bone marrow environment undergo morphological changes and become distinguishable from normal stem cells in a normal bone marrow environment by AI. Our results prompt us to provide a new area of research aiming to assess therapy response and predict disease outcomes by AI-aided identification and quantitation of normal stem cells in bone marrow of patients with stem cell-derived leukemias in the future.

We obtained our novel findings in this study successfully for two important reasons. Firstly, we took a transfer learning approach that helped to reduce the need for a large number of images and to make the model training process more efficient and correct. Secondly, we also took a multiple-CNN approach to allow achieving the highest classification accuracy using our AI-deep learning method. In current AI-related studies, a single-CNN approach is often taken based on arbitrary choice. In contrast, our multiple CNN approach is superior to the single-CNN approach in building more accurate AI models for analyzing cell images. In our multiple-CNN approach, we equally integrated 19 CNNs to ensure utilization of the strength of each CNN in order to reach the greatest potential of this technical approach. In addition, during the AI model building using the multiple CNNs, we included a model validation process that allowed for adjusting interactions of all CNNs involved prior to the completion of AI model building for use. On the other hand, we should point out that in our study, we paid much attention to the technical details in cell image preparation to ensure the consistency of our AI deep learning analysis of bone marrow cell morphology. Specifically, we used our automated cell stainer for fixing and staining all types of cells and used antibodies from the same manufacturers for all experiments. These efforts help to eliminate experimental variations that could undermine the conclusions drawn from our study.

Our ultimate goal is to use our AI deep learning method in medical practice, which would require our development of a cost-effective but efficient AI platform. Thus, we would like to point out that our multiple-CNN approach in building AI models and analyzing cell images require a regular computer with a compatible storage. Also, our multiple-CNN approach, compared to a single-CNN approach, ensures achievement of the highest classification accuracy. Furthermore, we emphasize paying much attention to the technical variations of cell images, which are often caused by several sources, including procedural differences in collecting and staining cells, types and settings of imaging machine/device, etc. Therefore, standardization of the methods for collecting cells and preparing cell images across hospitals is critical. In the future use of our AI method in medical practice, we envision that we could analyze any single-cell images from bone marrow of leukemia patients directly and obtain analytical results within hours, which can be done by using a portable computer with a regular capacity. The major challenge is the maintenance of the technical consistency when the single-cell images are prepared. Regardless, we believe that we are much closer to employing our AI deep learning method in patients for helping to guide therapeutic management of leukemia patients.

## 4. Materials and Methods

### 4.1. Mice

Conditional *JAK2V617F* knock-in mice in C57BL/6 background were kindly provided by Dr. Golam Mohi at University of Virginia, Charlottesville, VA, USA. After activation of *JAK2V617F* expression, these mice develop myeloproliferative disease [19,21]. Wild-type C57BL/6 littermates were produced during the breeding of *JAK2V617F* knock-in mice.

### 4.2. Single-Cell Image Preparation

Bone marrow cells from femurs and tabias of wild type or *JAK2V617F* knock-in mice treated with PiPc were flushed out, stained with antibodies in the Direct Lineage Cell Depletion Kit (Mitenyl Biotec, Charlestown, MA, USA, Cat# 130-110-470) and loaded onto the Auto MACS columns (Mitenyl Biotec, Charlestown, MA, USA, Cat# 130-021-101) placed on the Auto MACS machine to separate the lineage-negative (Lin^−^) cells from the lineage-positive cells (Lin^+^). Lin^−^ cells collected from the Auto MACS columns were stained with following antibodies for cell sorting by flow cytometry: Lin^−^Sca-1^+^c-Kit^+^CD150^+^CD48^−^ for LT-HSCs (long-term hematopoietic stem cells), Lin^−^Sca-1^+^c-Kit^+^CD150^−^CD48^−^ for ST-HSCs (short-term hematopoietic stem cells), and Lin^−^Sca-1^+^c-Kit^+^CD150^−^CD48^+^ for MPPs (multipotent progenitors). The sorted cells were spun onto glass slides for staining with Wright and Giemsa (W&G) using the automated Aerospray Hematology Pro Slide Stainer (Biomedical System, Logan, UT, USA) which automatically fixed and stained the cells on the slides. The stained cells were then scanned and cropped in Photoshop to form single-cell images for building AI models and further analysis with AI. The antibodies against CD150, CD48, Sca-1 and c-Kit for isolation of LT-HSCs, ST-HSCs and MPPs were purchased from eBioscience (Waltham, MA, USA). The antibodies against CD45.1 and CD45.2 were purchased from Pacific Blue. To purify lineage-negative cells, the antibody cocktail (Mitenyl Biotec) that allows for elimination of Lin^+^ mature cells expressing CD3, CD4, CD8, B220, Gr-1, Mac-1, or Ter119 was used. Throughout this study, we paid much attention to optimization of the experimental procedures. To minimize variations between experiments, we consistently fixed and stained cells with the automated Aerospray Hematology Pro Slide Stainer for all experiments and used all antibodies from the same manufacturer under the same experimental conditions. Moreover, in each experiment, we included all experimental groups, especially the control group, to further minimize experimental variations. 

### 4.3. Digital Cell Image Analysis

Because cell images usually require simple preprocessing before they can be classified, to use the images with an existing network, they should match the expected input size for that network. This varies depending on the network, but most pretrained networks expect a square color image around 220 pixels high by 220 pixels wide. As the cell images obtained are often different in size, our cropping and resizing of cell image were common processing steps when CNNs were used.

To get multiple CNNs to work together, the input image size must be resized to meet the various requirements of those CNNs before conducting training and testing (Table 1). We have prepared a stem cell dataset that contains a total of over 2000 individual single cell images for both training and testing, with about 80% of the images for training and about 20% for testing in establishing our AI deep learning models.

### 4.4. Transfer Deep Learning

Deep learning is a machine learning technique that uses deep neural networks with many layers to perform end-to-end learning, enabling the direct extraction of features and classification from raw input images. In other words, the images themselves are the inputs, and both feature extraction and image classification that are learned directly from the images. In our study, we classified cell images with CNNs that have already been trained on other image data. We customized these pretrained networks for analysis of our new single-cell images by modifying a pretrained network and retraining it on new, specific data to fit the desired classification task.

### 4.5. Algorithms and Training Options

The training algorithm was fine-tuned with many parameters, including the number of training images used at each step, the maximum number of iterations, the learning rate, etc. The training duration was adjusted by changing the number of epochs. At each iteration, a subset of the training images was used in the network, and CNNs were trained with optimized parameters to achieve reasonably good performance. These parameters included learning rate (0.001–0.0001), validation frequency (10–30), mini batch size (16–32), max epochs (20–100), and algorithms (sgdm, adam, and rmsprop). Once the network was trained on the whole training data set, one epoch had been completed.

### 4.6. Hardware and Software

The Matlab Deep Learning Toolbox was used to provide a framework for designing and implementing deep neural networks with algorithms, pretrained models, and apps. Image Processing Toolbox was used to provide a comprehensive set of reference-standard algorithms and workflow apps for image processing, analysis, visualization, and algorithm development. Matlab software (MATLAB R2021a) and a NVidia Titan XP GPU were used to train and test all networks in this study.

### 4.7. Code and Data Availability

The source code and datasets that were developed used in this study can be obtained from the corresponding author upon request to shaoguang.li@umassmed.edu.

## Figures and Tables

**Table 1:** The input image sizes of pretrained CNNs.

No.	Name of the Pretrained CNNs	Input Image Sizes (Pixels)
1	GoogleNet	224 × 224
2	Vgg16	224 × 224
3	Vgg19	224 × 224
4	ResNet18	224 × 224
5	ResNet50	224 × 224
6	ResNet101	224 × 224
7	MobileNetv2	224 × 224
8	DenseNet201	224 × 224
9	Places365GoogleNet	224 × 224
10	NASNetMobile	224 × 224
11	ShuffleNet	224 × 224
12	EfficientNetb0	224 × 224
13	AlexNet	227 × 227
14	SqueezeNet	227 × 227
15	DarkNet19	256 × 256
16	DarkNet53	256 × 256
17	Inceptionv3	299 × 299
18	Xception	299 × 299
19	InceptionResNetv2	299 × 299

## Data Availability

Original data related to this work can be obtained by contacting Dr. Shaoguang Li (shaoguang.li@umassmed.edu).

## Abbreviations

The following abbreviations are used in this manuscript:

PV	polycythemia vera
AI	artificial intelligence
CNN	convolutional neural networks
HSCs	hematopoietic stem cells
MPPs	multipotent progenitors
LSCs	leukemia stem cells
